# Sustained Delivery of the Antiviral Protein Griffithsin and Its Adhesion to a Biological Surface by a Silk Fibroin Scaffold

**DOI:** 10.3390/ma16165547

**Published:** 2023-08-09

**Authors:** Wenyan Guan, Ning Zhang, Arjan Bains, Airam Martinez, Patricia J. LiWang

**Affiliations:** 1 Materials and Biomaterials Science and Engineering, University of California Merced, 5200 North Lake Rd., Merced, CA 95343, USA; wguan3@ucmerced.edu; 2 Qingdao Institute of Bioenergy and Bioprocess Technology, Chinese Academy of Sciences, Qingdao 266101, China; zhangn2022@qibebt.ac.cn; 3 Chemistry and Biochemistry, University of California Merced, 5200 North Lake Rd., Merced, CA 95343, USA; abains5@ucmerced.edu; 4 Department of Bioengineering, University of California Merced, 5200 North Lake Rd., Merced, CA 95343, USA; amartinezrodriguez2@ucmerced.edu; 5 Molecular Cell Biology, Health Sciences Research Institute, University of California Merced, 5200 North Lake Rd., Merced, CA 95343, USA

**Keywords:** silk fibroin, HIV prevention, Griffithsin, drug delivery, adhesion

## Abstract

The protein Griffithsin (Grft) is a lectin that tightly binds to high-mannose glycosylation sites on viral surfaces. This property allows Grft to potently inhibit many viruses, including HIV-1. The major route of HIV infection is through sexual activity, so an important tool for reducing the risk of infection would be a film that could be inserted vaginally or rectally to inhibit transmission of the virus. We have previously shown that silk fibroin can encapsulate, stabilize, and release various antiviral proteins, including Grft. However, for broad utility as a prevention method, it would be useful for an insertable film to adhere to the mucosal surface so that it remains for several days or weeks to provide longer-term protection from infection. We show here that silk fibroin can be formulated with adhesive properties using the nontoxic polymer hydroxypropyl methylcellulose (HPMC) and glycerol, and that the resulting silk scaffold can both adhere to biological surfaces and release Grft over the course of at least one week. This work advances the possible use of silk fibroin as an anti-viral insertable device to prevent infection by sexually transmitted viruses, including HIV-1.

## 1. Introduction

Since the HIV pandemic began, about 84.2 million people have been infected with the HIV virus and 40.1 million people have died from it. A total of 38.4 million people were living with HIV in 2021, with 1.5 million new HIV infections worldwide every year [1]. HIV can be transmitted through sexual activity, blood transfusions, and parenteral exposure via needles, prenatally, and through breastmilk [2,3]. Most new infections appear in developing countries and, in most cases, affect women and young girls. In the United States, 68% of new HIV diagnoses in 2020 were among men who had sex with men; people who injected drugs, sex workers, and prisoners were also disproportionally affected by HIV [4,5].

HIV-1 is a member of the lentivirus family that is 100 nM in diameter, and it is covered with surface proteins composed of gp120 and gp41 [6]. Gp120 is the outer spike component, while gp41 is connected to the viral lipidic membrane [6]; these form trimeric glycoproteins on the surface of HIV-1 and are responsible for targeting viral entry into the cell [7]. Gp120 binds to its receptor, CD4, which is mainly expressed on the surface of the CD4 T cells, and this causes conformational changes in gp120 to facilitate the exposure of the fusogenic domain of the gp41 to start the viral fusion with the host membrane [8].

Scientists have been working on an HIV vaccine for more than 30 years. Unlike other viruses such as polio, smallpox, influenza, measles, and COVID, which can be effectively targeted by antibodies to produce long-term or even life-long immunity against subsequent infection, the immune system fails to produce an effective antibody response to the infection. HIV infects, disables, and kills CD4 T cells, which are crucial to the intact function of the immune system [9]. After infection, T-lymphocytes and other immune cells can then become latent reservoirs that harbor the virus and that may release HIV at any time. Therefore, even though current small molecule drugs can essentially eliminate the circulating virus, if medication is stopped, HIV reservoirs can be activated, and viremia will rebound. Furthermore, HIV mutates rapidly, which leads to changes in the structure of proteins that would normally be the target of antibodies, allowing HIV to elude the immune system [9]. Most recently, the failure of an HIV vaccine in a Phase 3 clinical trial was announced by a pharmaceutical company, Johnson & Johnson, on 18 January 2023 [10].

After four decades of endeavors, multiple treatments have been approved by the FDA (Food and Drug Administration), including antibodies [11], protease inhibitors [12], nonnucleoside reverse-transcriptase inhibitors [12], integrase inhibitors [12], and combination antiretroviral therapy with three or more drugs treatment regimens (cART) [13]. These prolong a patient’s life, but there is still no cure and no vaccine currently available [13]. Therefore, preventing HIV infection in uninfected and/or at-risk populations is vitally important. More than 10 years ago, the FDA approved the first PrEP (pre-exposure prophylaxis) drug Truvada, composed of emtricitabine and tenofovir disoproxil fumarate, both of which are nucleotide reverse transcriptase inhibitors (NRTI), to reduce the risk of HIV infection among high-risk populations and among those who may engage in sexual activity with HIV-infected partners [14]. It has been found that using a daily PrEP by at-risk but uninfected people reduces the sexual acquisition of HIV infection by 99% [15]. Daily oral PrEP, however, relies heavily on a person’s ability to adhere to an active prescription or proper schedule, which can be problematic in some settings, particularly for women and girls in the developing world [16]. There is also a concern regarding increasing viral drug resistance when antiretroviral drugs for PrEP are also used as an actual treatment for infected people [17].

As an alternative to a daily pill, microbicides can be useful as a tool to prevent HIV infection. Microbicides are a type of product or substance that could be inserted into the vaginal or rectal tract to safely prevent HIV acquisition through sexual transmission [18]. Microbicides can be applied to deliver an anti-HIV drug (PrEP product) topically to the vaginal or rectal mucosa surface through materials including foams, films, vaginal rings, gels, etc. [19]. The application of such microbicides provides users with a protection that they can control. Therefore, microbicides have been actively studied and developed as a potential tool in the application against sexually transmitted infections of HIV, especially in resource-poor areas where access to other prevention approaches including condoms or oral PrEP may be limited. An ideal microbicide should be biocompatible, nontoxic, cheap, easy to apply, highly potent against HIV, potentially able to mediate sustained release of the anti-HIV drug, and not require refrigeration. The silk fibroin based anti-HIV drug delivery system has all of these properties, making it an excellent candidate for microbicide formulation.

Silk fibroin (SF or “silk” used herein) is derived from silkworm (*Bombyx mori*) cocoons and is a high-molecular-weight natural protein polymer that has been recognized as a material for biomedical applications for centuries. Silk-based sutures were approved by the FDA in 1993 [20,21]. Since then, four silk-based biomedical materials have been commercialized, including bioresorbable surgical mesh (SERI Surgical Scaffold^®^) [22], silk fabric (MICROAIR DermaSilk^®^) for atopic dermatitis in children [23,24], a silk patch (Tympasil) for acute tympanic membrane perforation treatment [25], and a silk fibroin wound dressing (Sidaiyi) [26]. With its superior biocompatibility, controllable biodegradation into noninflammatory byproducts, aqueous processibility, compatibility with sterilization, robust mechanical and thermal properties, and sufficient supply, silk has become a popular biomaterial for drug delivery, as a biosensor, and for tissue engineering [27,28,29,30,31,32,33,34,35,36,37]. It has been shown that silk is able to encapsulate a variety of molecules, including protein HIV inhibitors, antibodies, and small molecules, with multiple modalities such as 3D porous scaffolds, films, gels, particulates, and microneedle patches, contributing a desired drug delivery system as a microbicide [38,39,40,41].

We have shown that release of the HIV inhibitor Griffithsin (Grft) in silk discs in vitro can be sustained for 1 month and that Grft remains functional for 14 months at 25 °C, 37 °C, and 50 °C [38]. Grft is a lectin derived from red algae that can tightly bind to high-mannose glycosylation sites on viral surfaces. In HIV, Grft binds the envelope glycoprotein gp120 to inhibit viral infection and is one of the most potent lectin inhibitors of HIV [42,43,44]. It has also exhibited effective inhibition of other enveloped viruses such as SARS (SARS-CoV-1 and 2) [45,46], and Hepatitis C [47], as well as the human papillomavirus (HPV) [48], Japanese encephalitis virus (JEV) [49], and Hantaan virus (HTNV) [50]. When Grft is encapsulated into silk fibroin, the SF discs show strong inhibition properties in activated peripheral blood mononuclear cells (PBMC) and human colorectal and cervical tissue explants. In macaques, insertable SF–Grft disks protected both vaginal and rectal tissue from SHIV infection ex vivo without triggering inflammatory cytokine secretion [38,51]. However, the weak adhesion of silk film discs to tissue surfaces may hinder their application in clinical usage.

The objective of this study was to improve the SF discs’ adherence to biological tissue while retaining sustained release of the protein drug. Four chemical additives, tannic acid; Tren-Lys-Cam (TLC: tris(2-aminoethyl)amine-glycyl-2,3-dihydroxy-benzamide, a siderophore analogue); 3,4-dihydroxybenzoic acid (3,4-DHB); and hydroxypropyl methylcellulose (HPMC), which may improve SF disc adhesion to the tissue, were formulated into SF discs. It was found that the Silk–HPMC disc can adhere to sample tissue, but this was also accompanied by swift disintegration of the film (within 30 min). Therefore, various additives were incorporated into the silk films, including polyethylene glycol (PEG), glucose, and glycerol, to slow down the disintegration of the Silk–HPMC disc. The results show that the addition of glycerol can decrease the disintegration of the Silk–HPMC while retaining the adhesion and sustained release of Grft. Finally, in vitro studies showed that the Grft released from Silk–HPMC–Glycerol–Grft discs remained active against HIV.

## 2. Materials and Methods

### 2.1. Production of Grft

The protein Griffithsin was prepared as described previously, with small modifications [52,53]. Briefly, the gene encoding the protein Grft with an N-terminal His6 tag in a pET15b vector was transformed into *E. coli* BL21(DE3) cells (Novagen, Madison, WI, USA) and cultured in Luria–Bertani (LB) medium (VWR, Avantor, Radnor, PA, USA). After expression was induced by the addition of 0.5 mM IPTG (Gold Biotechnology, Olivette, MO, USA) for 4–6 h at 37 °C, cells were harvested by centrifugation at 4200× *g*. The cell pellet was then resuspended in lysis buffer (6 M guanidinium chloride (VWR), 200 mM NaCl (Thermo Fisher Scientific, Waltham, MA, USA), 50 mM Tris (Thermo Fisher Scientific) pH 8.0) followed by sonication (Qsonica, Newtown, CT, USA) for 10 min to disrupt the cells. After centrifugation, the supernatants were collected and purified by a home-packed Ni-NTA affinity column (Qiagen, Hilden, Germany). The protein was eluted with a pH gradient ending with pH 4.0, which was then added dropwise at a 10-fold dilution into refolding buffer (550 mM L-arginine hydrochloride, 200 mM NaCl, 50 mM Tris, 1 mM EDTA (Thermo Fisher Scientific), pH 8.0) and allowed to refold for 24 h at 4 °C while stirring. Then, the protein was dialyzed twice against 4 L of buffer A (200 mM NaCl, 20 mM Tris pH 8.0) at 4 °C and then twice against 4 L of buffer B (80 mM NaCl, 20 mM Tris pH 8.0) at 4 °C. Finally, the folded Grft was further purified on a C4 reversed-phase chromatography column (Vydac, Hesperia, CA, USA) with an acetonitrile gradient. The fractions containing pure Grft, as determined by SDS-PAGE, were combined and lyophilized.

### 2.2. Production of HIV-1 Pseudovirus

The single-round virus used contained a lentiviral core with the envelope protein of the HIV strain PVO.4 on its surface and was a kind gift from Dr. Kathryn Fischer [42]. The viral plasmids’ env gene from HIV-1 was from the AIDS Research and Reference Reagent Program, Division of AIDS, NIAID, NIH, whose details are as follows: PVO, clone 4, strain SVPB11 (referred to as PVO.4), from David Montefiori and Feng Gao [54]. TZM-bl cells were obtained from the NIH AIDS Research and Reference Reagent Program, Division of AIDS, NIAID, NIH from Dr. John C. Kappes, Dr. Xiaoyun Wu, and Tranzyme Inc. (Durham, NC, USA) [55,56,57,58]. The TZM-bl cells were cultured in DMEM (Gibco, Life Technologies, Carlsbad, CA, USA) containing 10% FBS, 25 mM HEPES (Thermo Fisher Scientific), 1.1% GlutaMAX (Gibco), 0.3% Penicillin/streptomycin (Antibiotic-Antimycotic Solution, Cellgro, Lincoln, NE, USA).

### 2.3. Preparation of Silk Fibroin Solution

The SF isolation and solution preparation is the same as described previously [38,39,59]. Briefly, *Bombyx mori* cocoons were cut into about 1 cm^2^ pieces and inspected for debris and stains. Clean cocoon pieces were degummed by boiling in 20 mM Na_2_CO_3_ (Thermo Fisher Scientific) for 30 min to remove the sericin protein. The fibers were then rinsed thoroughly in deionized water and air-dried for 2 days. The dried fibers were then dissolved in 9.3 M LiBr (Thermo Fisher Scientific) (1 g silk fibroin fibers in 4 mL LiBr solution) for 4 h at 60 °C followed by dialysis against deionized water for 4 days to remove LiBr. The resulting silk solution was centrifuged to remove debris, and the solution was sterilized by autoclaving. The SF stock solution was determined to be 5.89% (*w*/*v*) and was then stored at 4 °C until use. The concentration of SF using this procedure tends to range from 4–6% (*w*/*v*).

### 2.4. Production of Silk-Based Discs

The SF stock solution (5.89%) was used to prepare all silk-based discs in this study. The Grft powder was dissolved in 20 mM HEPES (Thermo Fisher Scientific) pH 8.0 buffer, and the concentration was determined by absorbance at 280 nm.

For chemical additives used for increasing adhesion, we tested 4 candidates: tannic acid (VWR), TLC, 3,4-DHB, and HPMC (Thermo Fisher Scientific). The TLC and 3,4-DHB were a gift from Prof. Roberto Andresen Eguiluz and Dr. Syeda Tajin Ahmed. The Silk–Tannic acid scaffold solution was composed of 1% tannic acid and 2% silk. The Silk–TLC scaffold solution was made at two concentrations: 50 μM TLC with 2% silk, and 500 μM TLC with 2% silk. The Silk–3,4-DHB scaffold solution also was made at two concentrations: 50 μM 3,4-DHB with 2% silk and 500 μM 3,4-DHB with 2% silk. The Silk–HPMC scaffold solution was made with three formulations: 0.75% HPMC with 2% silk, 1.5% HPMC with 2% silk, and 3% HPMC with 2% silk. All these scaffold solutions were aliquoted to 200 μL in 1.5 mL centrifuge tubes and frozen at −80 °C for 2 h followed by lyophilization to make the scaffold.

To determine the best candidate for decreasing the disintegration of Silk–HPMC discs, we tested 3 additives: PEG (polyethylene glycol 400, Merck KGaA, Darmstadt, Germany), glucose (Thermo Fisher Scientific), and glycerol (Thermo Fisher Scientific). The Silk–HPMC–PEG scaffold solution was made up of 1% PEG, 3% HPMC, and 2% silk. The Silk–HPMC–glucose scaffold solution was made up of 15% glucose, 3% HPMC, and 2% silk. The Silk–HPMC–glycerol scaffold solution was made up of 1% glycerol, 3% HPMC, and 2% silk. All these scaffolds’ solutions were aliquoted to 1 mL in 24-well plates followed by using one of two processing conditions. The first condition (“condition 1”) was to cool the samples to −80 °C and then freeze-dry the samples, followed by annealing in an 80 °C water bath for 4 days. The second condition (“condition 2”) was to anneal the samples in an 80 °C water bath for 1 h to allow HPMC polymerization; the samples were then cooled to −80 °C and freeze-dried, followed by annealing in an 80 °C water bath for 4 days. All samples were dried in a 50 °C incubator for 2 h after the processing mentioned above. This results in a flat cylindrical disc of diameter 1.6 cm. The thickness is 0.4 cm for discs made with 1 mL silk-based solution, and 0.8 cm for those in the silk adhesion tests (below) that were made with 2 mL of the silk-based solution.

The discs for the sustained release of Grft experiments include Silk–Grft, Silk–HPMC–Grft, and Silk–HPMC–Glycerol–Grft. The Silk–Grft scaffold solution was made up of 10 μM Grft and 2% silk. The Silk–HPMC–Grft scaffold solution was made up of 10 μM Grft, 3% HPMC, and 2% silk. The Silk–HPMC–Grft scaffold solution was made of 10 μM Grft, 3% HPMC, 1% glycerol, and 2% silk. All these scaffolds’ solutions were aliquoted to 1 mL in 24-well plates followed by condition 2 processing (see above). All samples were dried in a 50 °C incubator for 2 h after the processing. To sterilize the samples for the Grft release experiments (because Grft was later used in pseudoviral assays with mammalian cells), the discs were placed in a biosafety cabinet with the UV light on for 1 h before adding PBS.

The discs for the tissue adhesion test, Silk, Silk–HPMC, Silk–HPMC–Glycerol, and Silk–HPMC–Glycerol–Grft, were made in the same way as the discs for the sustained release of Grft experiments, except that each disc was made in 2 mL of solution in 24-well plates to allow for a thicker disc that can be more easily tested with hanging weights.

The discs for the degradation experiment were made under processing condition 2, including 2%Silk–3%HPMC, 2%Silk–3%HPMC–1%PEG, 2%Silk–3%HPMC–15%Glucose, 2%Silk–3%HPMC–1%Glycerol, and 2%Silk–3%HPMC–1%Glycerol–10 μM Grft.

For FTIR experiments, the unannealed discs were of the same formula solution as the discs for the sustained release of Grft experiments followed with freeze-drying without annealing (that is, without further processing in a water bath or being placed in a warm incubator). The annealed discs were made in the same way as the discs for the sustained release of Grft experiments.

The colorful silk-based discs were made by adding small amounts of green, red, blue and yellow food coloring (McCormick, Hunt Valley, MD, USA) to the silk solution and placing it into shaped molds rather than 24-well plates, followed by lyophilization with no further processing.

### 2.5. Morphology of Silk-Based Discs

The interior morphology of the silk-based discs was assessed by scanning electron microscopy (SEM) using a Zeiss EVO MA10 electron microscope (Carl Zeiss AG, Oberkochen, Germany). The samples were cut to expose the cross-sections, mounted onto SEM stubs, and coated with gold via a SC7620 sputter coater (Quorum Technologies, Lewes, UK). The diameter of the pore in each image was measured by the average of 20 pores in ImageJ (version 1.53).

Fourier-transform infrared spectroscopy (FTIR; JASCO FTIR 6200 spectrometer, Jasco, Easton, MD, USA or a Bruker Vertex 70 spectrometer, Berlin, Germany) was used to evaluate the secondary configurations of silk-based discs, as described previously [38]. The amide I region (1585 to 1720 cm^−1^) was overlaid to compare the β-sheet content.

### 2.6. Adhesion Tests

The preliminary adhesion tests were carried out by placing a silk-based disc onto the wet surface of the sample skin (chicken skin from commercial sources) and using a tweezer to lift the disc to see whether the disc could adhere well enough to draw up the tissue when pulled (Supplementary Video S1). If it was able to pull up the tissue, then it was considered a candidate for further experiments.

To measure the adhesion differences among the 4 SF-based discs: Silk, Silk–HPMC, Silk–HPMC–Glycerol, and Silk–HPMC–Glycerol–Grft, the retention time that a disc could remain attached to the tissue under a pulling force was recorded. First, the tissue was rinsed and submerged in PBS for 5 min, and it was then stabilized horizontally against a glass surface (measuring 10 cm × 7.4 cm). An SF disc was placed on the tissue with brief finger pressure, then incubated for 5 min and allowed to adhere, followed by rotating the set-up to a vertical orientation. Under these conditions, it was found that the discs were able to adhere indefinitely. Therefore, a further test of adherence to the skin was carried out using small weights (12 g total) attached to the disc. The length of time that a disc retained its hold on the tissue was measured.

### 2.7. Measurement of Sustained Release of Grft from the SF Discs

The Grft released from 3 SF-based discs, namely Silk–Grft, Silk–HPMC–Grft, and Silk–HPMC–Glycerol–Grft, were measured by BLI (biolayer interferometry) (Gator Bio, Palo Alto, CA, USA). To test for Grft release, 1 mL of PBS was added into each disc-containing well (of the 24-well plate) and incubated in a 37 °C incubator. The initial “burst” effect of release was collected after the first hour by removal of the PBS, and the solution was replaced with fresh PBS. Later timepoints were obtained by repeating this process daily for 7 days, each time collecting the PBS solution and replacing it with fresh PBS.

The concentration of Grft from these timepoints was then determined by a BLI quantitative assay. First, a standard curve was made, using pure Grft that had not been in a disc. For this, several standard Grft concentration solutions were prepared in PBS at pH 7.4. Four standard concentration solutions were measured by BLI and compared with two sustained release samples and one PBS buffer at the appropriate pH as a control. All samples were centrifuged for 3 min at 15,000 rpm to remove any debris before measurement. The pH of samples that were collected by soaking at pH 4.0 was adjusted to pH 7.4 to allow the His6-tag on Grft to be in a protonated state to bind to the BLI anti-His-tag sensor. Each type of disc was measured in triplicate with three separate samples.

### 2.8. HIV Inhibition Assays

The inhibition of Grft in the sustained release solutions mentioned in Section 2.7 (above) was tested for the effect on its activity against PVO.4 infection of TZM-bl cells [38,39] In brief, 6000 TZM-bl cells were seeded in a 96-well plate (100 μL per well) and incubated for 16 h. Then, the medium was removed and 20 μL of HIV-pseudovirus PVO.4 was added. For no-virus control wells, 20 μL/well of cell medium were added. Then, 15 μL of medium and 15 μL of the Grft sample was added. For negative control wells, 15 μL of medium and 15 μL/well of pH 7.4 PBS were added. After 10 h infection, 160 μL of media was added into each well and incubated at 37 °C for another 36 h.

The viral infection in TZM-bl cells was measured by luciferase quantification of cell lysates using the Bright-Glo Luciferase Assay System (Promega, Madison, WI, USA). In brief, most of the media was removed, leaving 20 μL in each well; then, 20 μL of Bright-Glo Luciferase Assay System solution was added and allowed to react for 3 min. After that, the cells and solution were transferred into a white 96-well plate (OptiPlate-96, PerkinElmer, Valencia, CA, USA) and incubated at room temperature for 5 min followed by the reading of the luminescence signal on a CLARIOstar plate reader (BMG Labtech, Cary, NC, USA).

### 2.9. Biocompatibility Assays

The biocompatibility of the Silk–HPMC–Glycerol–Grft disc was assessed by an MTT assay. In brief, HEK 293Ft cells (Homo sapiens, embryonic kidney cells; a gift from Dr. David Gravano, University of California, Merced, CA, USA) were seeded into a 96-well plate at a density of 10,000 cells per well with 90 μL of culture media and 10 μL of sample (the day 6 sustained release solutions mentioned in Section 2.7) or pH 7.4 PBS (control). The cells were cultured at 37 °C for 24 h. After that, the cell viability was measured by following the protocol of the MTT Cell Proliferation Assay Kit (Cayman Chemical, Ann Arbor, MI, USA).

### 2.10. Degradation Evaluation

The degradation of the silk-based discs, including Silk–HPMC, Silk–HPMC–Glycerol, Silk–HPMC–Glycerol–Grft, Silk–HPMC–PEG, and Silk–HPMC–Glucose, were measured by immersing the discs in a 24-well plate with 2 mL of PBS (pH 7.4 or both pH 7.4 and pH 4.0 for Grft-containing discs) in each well and incubated at 37 °C for 7 days. After that, each disc was placed in a 2 mL centrifuge tube and spun down for 10 min at 15,000 rpm. Then the supernatant was removed, and the pellets were vacuum-dried using a speed-vac at 60 °C for 5 h. The remaining dry weight (%) is an indicator of each disc’s extent of degradation. Silk–HPMC–Glycerol–Grft disc degradation experiments were performed in triplicate in both pH 7.4 and pH 4.0 PBS. Other discs’ degradation experiments were performed in duplicate in PBS pH 7.4.

### 2.11. Statistical and Mathematical Analysis

One-way and two-way ANOVA (Excel 365) were applied to evaluate the differences among several groups. The Student’s *t*-test (Excel 365) was utilized to compare two groups’ differences. Both the ANOVA and Student’s *t*-test alpha values were 0.05. A probability *p*-value less than 0.05 was considered statistically significant; NS indicates *p* > 0.05; * indicates *p* < 0.05; and ** indicates *p* < 0.01.

## 3. Results

### 3.1. HPMC Increases the Adhesion of the Silk Discs to Sample Tissue

To test adhesion in a biological system, moist animal skin was used. The biological tissue used in this study was chicken skin from Cornish Cross chickens. While not as close a mimic to human skin as porcine skin [60,61], chicken skin has both an epidermis and a dermis (like human skin) and has several similar components, including collagen, elastin, and keratinocytes [62,63]. Additionally, the advantage of being inexpensive and available commercially in large amounts makes it a good candidate for preliminary studies. A simple formulation of SF and Grft can adhere to a surface containing the skin, but its adhesion is not strong, as evidenced by its easy removal from the surface with a tweezer (Supplemental Video S1). Therefore, four candidates, tannic acid, TLC, 3,4-DHB, and HPMC, were blended with silk solution individually to form discs, and each disc (2% final SF) was tested for adhesion to the tissue. The silk films that included tannic acid showed significant precipitation on the side of the well and did not form sponge-like discs, so tannic acid was judged to be an unacceptable additive for this work. Discs made of SF/TLC and SF/3,4-DHB were tested at two concentrations of the additive (50 μM and 500 μM). While these did form sponge-like discs, none of them were able to adhere to moist skin that had been hydrated with either pH 7.4 or pH 4.0 PBS. Finally, discs were formulated with 3% HPMC and 2% silk fibroin in a total of 1 mL solution. This formed a disc that adhered well to skin (Supplementary Video S1). Lower HPMC concentrations were also used (1.5% and 0.75%), but these showed less ability to adhere to the tissue, so 3% HPMC was used in further experiments.

### 3.2. Glycerol Decreases the Disintegration of Silk–HPMC Discs

**Figure 1:**
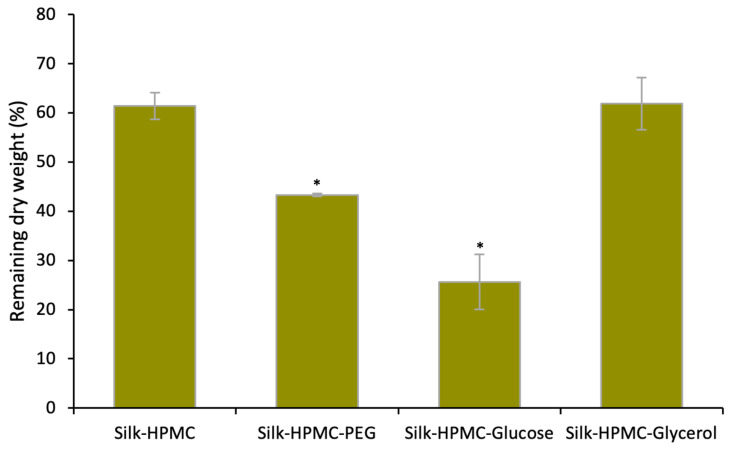
**The remaining dry weight (%) from a degradation experiment of silk-based discs.** Discs were immersed in PBS pH 7.4 for 7 days at 37 °C. Silk–HPMC–Glycerol showed the combined properties of lower degradation and a better ability to hold its shape. Two-way ANOVA showed significant differences among the groups. The Student’s *t*-test was applied to evaluate the extent of differences between Silk–HPMC and other discs. * indicates *p* < 0.05.

While 2% silk–3% HPMC discs adhered well to model skin samples, these discs did not hold their shape, and under some conditions (such as when silk discs were not annealed), they disintegrated on the wet tissue in 30 min. This property would be favorable in a situation where rapid drug release and quick protection are a priority. However, this would not be ideal for use as a sustained release device to allow several days or weeks of protection. Therefore, additives were sought to slow the degradation of the film. Three candidates, PEG 400, glucose, and glycerol, which have been reported to increase the crystallinity of the silk scaffold [64,65], were used. In this study, for 1 mL working solution, the final silk concentration was 2%, and the final HPMC concentration was 3%. The candidates’ concentrations were (separately) PEG 1%, glucose 15%, and glycerol 1%. The results showed that over the course of a week of incubation in PBS pH 7.4, Silk–HPMC–Glycerol scaffolds held their shape and degraded (as judged by the remaining dry weight) approximately as much as Silk–HPMC scaffolds. Both of these showed less degradation than Silk–HPMC–PEG and Silk–HPMC–Glucose scaffolds (Figure 1). Therefore, Silk–HPMC–Glycerol was judged to have desirable properties for further study.

### 3.3. The Morphology and FTIR Spectra of the Silk Discs

**Figure 2:**
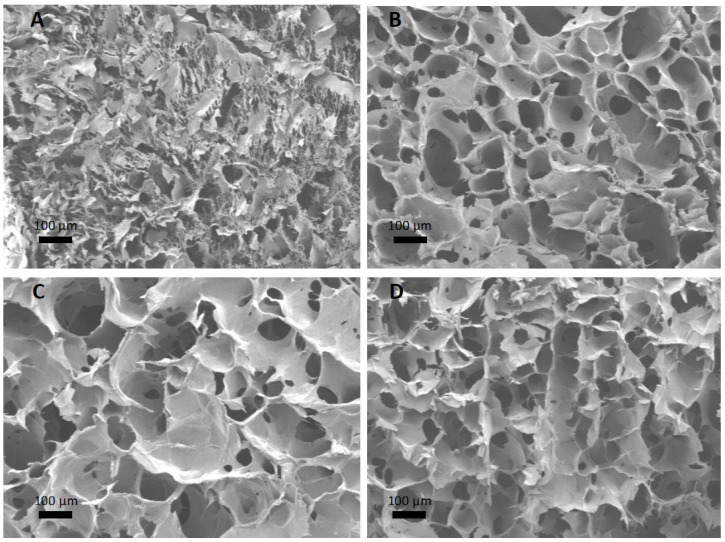
**SEM images of the 4 silk-based scaffolds.** (**A**) Control silk disc. (**B**) Silk–HPMC disc. (**C**) Silk–HPMC–Glycerol disc. (**D**) Silk–HPMC–Glycerol–Grft disc. Pore size was measured with ImageJ.

The four silk-based discs, Silk, Silk–HPMC, Silk–HPMC–Glycerol, and Silk–HPMC–Glycerol–Grft, processed under condition 2, were cut to expose the internal structure and evaluated via scanning electron microscopy (SEM; Figure 2A–D). The control Silk disc (with no additives) has smaller pores with diameter 20 ± 9 µm in its structure compared with the other three types of discs. Only small visual differences were observed among the Silk–HPMC (diameter 68 ± 30 µm), Silk–HPMC–Glycerol (diameter 120 ± 70 µm), and Silk–HPMC–Glycerol–Grft (diameter 82 ± 60 µm) discs.

**Figure 3:**
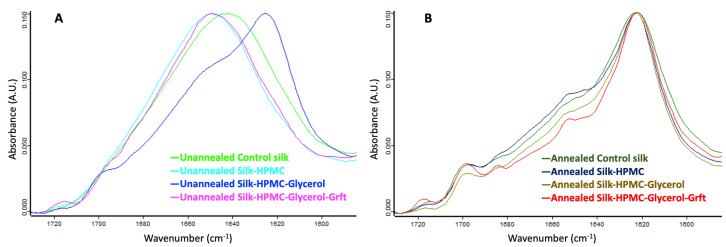
**FTIR spectra of 4 unannealed and annealed silk-based scaffolds**. (**A**) Unannealed scaffolds: control silk (light green), Silk–HPMC (light blue), Silk–HPMC–Glycerol (blue), and Silk–HPMC–Glycerol–Grft (magenta). (**B**) Annealed scaffolds: control silk (green), Silk–HPMC (dark blue), Silk–HPMC–Glycerol (brown), and Silk–HPMC–Glycerol–Grft (red). Unannealed refers to SF scaffolds that are not further processed beyond lyophilization. Annealing refers to processing under humid conditions as described in Section 2.4 of the Methods (condition 2).

FTIR was performed to analyze the protein secondary structure of the four silk-based discs (Figure 3A,B). For the four unannealed (no processing after simple lyophilization) silk-based discs, Silk–HPMC–Glycerol has a higher β-sheet content (wavenumber 1619–1628 and 1697–1703 cm^−1^) [66] than the other three discs. After processing under condition 2, however, the four annealed silk-based discs all transformed into more of a β-sheet structure to form a silk II assembly, which is an organized crystalline form of fibroin that can slow down the degradation of the discs [66].

### 3.4. Adhesion of the Four Silk-Based Discs

**Figure 4:**
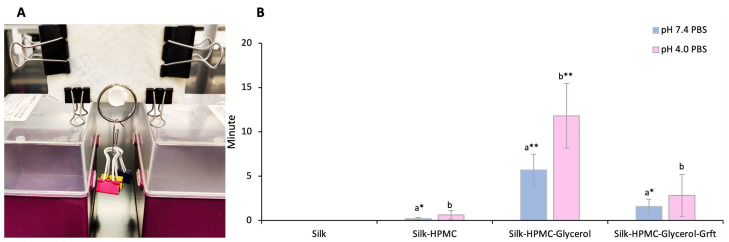
**Adhesion test of the 4 silk-based scaffolds**. (**A**) The set up of the adhesion test. A silk disc was placed on chicken skin that was stabilized on a glass surface and then oriented perpendicular to the table. All silk discs were able to remain on the skin for at least 2 h under these conditions. To further test adherence to the biological surface, a weight was added to the disc (a ring and three clips connected to the ring). The time was measured until the silk disc fell from the biological surface. (**B**) The time that various formulations of silk disc could remain adhered to the surface in the presence of a 12 g weight; pH 7.4 (blue) and pH 4.0 (pink). Two-way ANOVA shows significant differences among groups. The Student’s *t*-test was applied to evaluate the extent of differences between different groups: a—significance between silk and other discs at pH 7.4; b—significance between silk and other discs at pH 4.0. There was no significant difference for each disc between pH 7.4 and pH 4.0. * indicates *p* < 0.05; ** indicates *p* < 0.01. The *p*-value for Silk–HPMC–Glycerol–Grft compared to Silk alone is 0.1 due to the large variation in the adhesion time.

Adhesion of the four silk-based discs, Silk, Silk–HPMC, Silk–HPMC–Glycerol, and Silk–HPMC–Glycerol–Grft, to the tissue (chicken skin) was tested under two pH conditions, pH 7.4 and pH 4.0. These pH values were chosen to mimic the pH environment of the rectal area and the vaginal area, respectively. Each type of disc was prepared from 2 mL solution with condition 2 processing and was measured in triplicate with three separate samples. The tissue was washed and soaked with PBS, and then clamps were used to stabilize the tissue on a horizontal glass surface. Then, one of the silk discs was placed on the tissue for 5 min (to approximate a user inserting the disc and then carrying out an activity such as walking soon thereafter), and the surface was rotated to a vertical orientation. Under these conditions, each silk film adhered to the surface indefinitely. Because actual use may include various forces on the insert, small weights were added to determine the robustness of adherence (total weight 12 g). The length of time that the disc was able to retain its hold on the skin with weights attached was measured (Figure 4A). The control silk discs had no retention time, falling off the tissue immediately at both pH 7.4 and pH 4.0. The Silk–HPMC discs did adhere longer, falling after 13 ± 7.6 s at pH 7.4 and 38 ± 28 s at pH 4.0, on average. The weighted Silk–HPMC–Glycerol discs adhered to the vertical surface most tightly, attaching to the tissue for 320 ± 86 s at pH 7.4 and 710 ± 220 s at pH 4.0, on average. The Silk–HPMC–Glycerol–Grft discs showed good adherence, being able to maintain their position on the vertical surface while weighted for 96 ± 48 s at pH 7.4 and 170 ± 140 s at pH 4.0, on average.

### 3.5. Silk–HPMC–Glycerol–Grft Discs Show Sustained Release of Griffithsin

**Figure 5:**
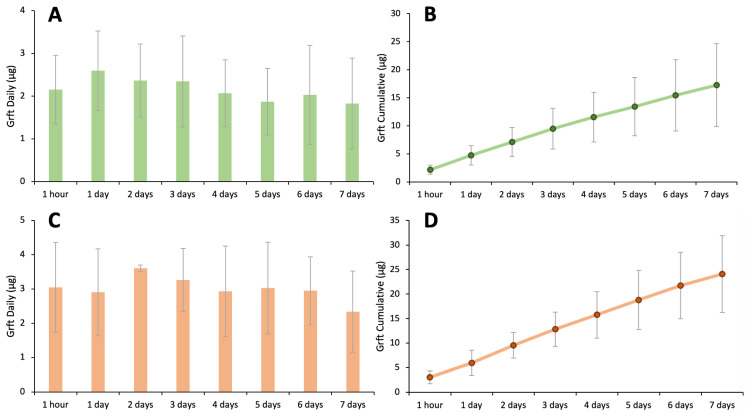

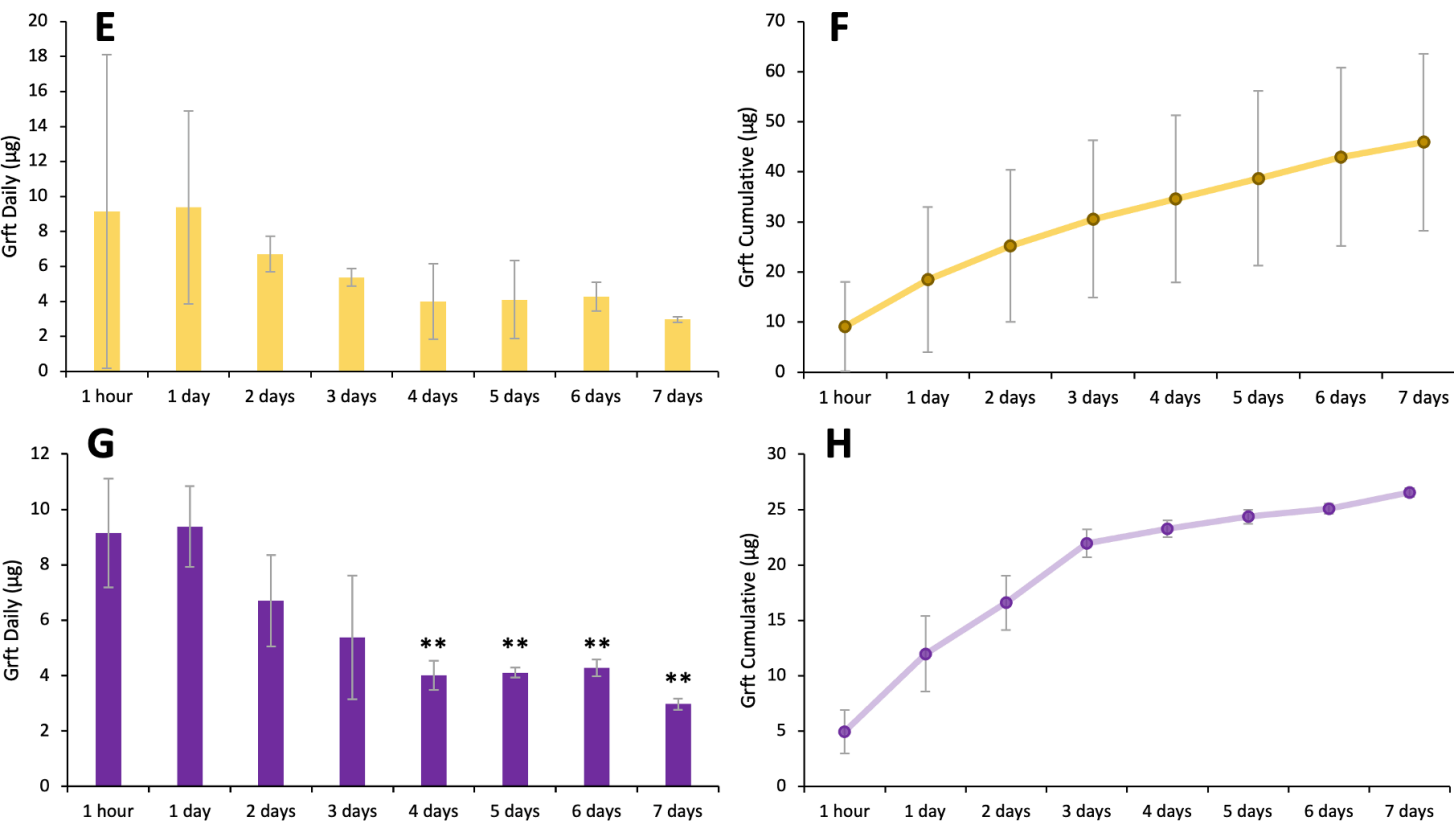
**Sustained release of Grft from silk-based disks**. (**A**) The sustained release of Grft from Silk–HPMC–Glycerol–Grft disks in pH 7.4 PBS. (**B**) The accumulated release of Grft from Silk–HPMC–Glycerol–Grft disks in pH 7.4 PBS. (**C**) The sustained release of Grft from Silk–HPMC–Glycerol–Grft disks in pH 4.0 PBS. (**D**) The accumulated release of Grft from Silk–HPMC–Glycerol–Grft disks in pH 4.0 PBS. (**E**) The sustained release of Grft from Silk–HPMC–Grft disks in pH 7.4 PBS. (**F**) The accumulated release of Grft from Silk–HPMC–Grft disks in pH 7.4 PBS. (**G**) The sustained release of Grft from Silk–Grft disks in pH 7.4 PBS. (**H**) The accumulated release of Grft from Silk–Grft disks in pH 7.4 PBS. All discs were processed under condition 2 (Methods Section 2.4). No significant differences were observed in the results of (**A**,**C**,**E**) evaluated over the 7-day experiment by one-way ANOVA. For (**G**), significant differences were observed among the timepoints, and the Student’s *t*-test was applied to evaluate the extent of the differences between the 1-day timepoint and the other timepoints; ** indicates *p* < 0.01.

It is important that a disc formulated with Grft retains its ability to release Grft when used. Furthermore, while some users may prefer immediate protection from the insertion of a disc, the key benefit of using a biocompatible, non-immunogenic, naturally-sourced material for the discs (i.e., silk fibroin) is that it can be well tolerated over several days of continuous contact with the human body and can sustained release therapeutics over a certain period of time. Therefore, the discs were tested to determine the level of Grft released. For these experiments, biolayer interferometry (BLI) was used. This is a relatively facile technique that can monitor the amount of a protein that binds to a solid support by measuring the change in wavelength of light shone through the probe upon binding. Since Grft retains a 6-His tag (which has been shown to not affect its activity), the protein readily binds to a commercial BLI sensor. BLI was used to evaluate the sustained release of Griffithsin from the three silk-based discs, including Silk–Grft, Silk–HPMC–Grft, and Silk–HPMC–Glycerol–Grft. (When tested for degradation, Silk–HPMC–Glycerol–Grft discs retained 46% of their dry weight after 1 week; the same discs without Grft retained 62% of their dry weight under the same conditions. Discs containing PEG or glucose were not considered because those additives did not slow the degradation of the disc, as shown in Figure 1). Each type of disc encapsulated 147 μg Grft (10 μM final concentration) in 1 mL of silk scaffold solution followed by condition 2 processing to formulate the discs. All experiments were carried out in triplicate. The silk discs were soaked in pH 7.4 and pH 4.0 PBS solutions to represent colorectal and vaginal conditions, respectively. At various timepoints, the incubation solution was removed and replaced with fresh PBS (again at pH 7.4 and pH 4.0, respectively), and the Grft released from the discs was measured (Figure 5A–H). No obvious burst release of Grft in the 1st hour was observed in any of the discs: Silk–HPMC–Glycerol–Grft (2.2 μg (147 nM) at pH 7.4 and 3.0 μg (207 nM) at pH 4.0, representing ~2% of the total amount of Grft loaded); Silk–Grft discs (4.9 μg (336 nM), representing ~3%); and Silk–HPMC–Grft discs (9.2 μg (623 nM), representing ~6%). Rather, in each case, steady release was observed. The accumulated release of Grft in Silk–HPMC–Glycerol–Grft discs after 7 days was ~17 μg in pH 7.4 PBS and ~24 μg in pH 4.0 PBS. The accumulated release of Grft in Silk–HPMC–Grft discs after 7 days was ~46 μg, while it was ~27 μg in Silk–Grft discs in pH 7.4 PBS.

### 3.6. The Griffithsin Released from the Silk–HPMC–Glycerol–Grft Discs Shows Inhibitory Activity against HIV In Vitro

**Figure 6:**
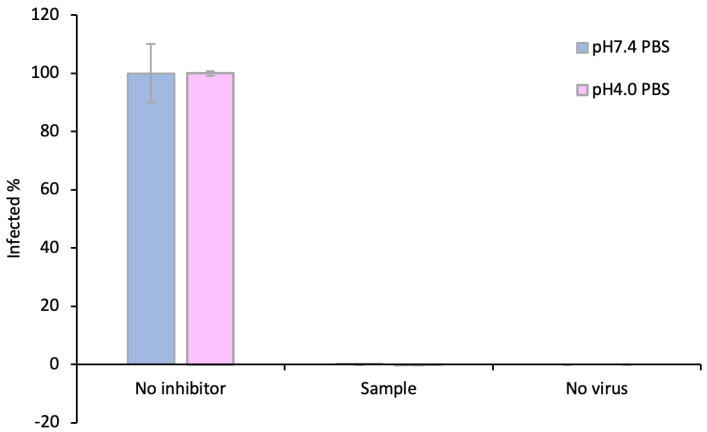
The Grft released from Silk–HPMC–Glycerol–Grft disks showed strong inhibition against HIV at both pH 7.4 and pH 4.0 PBS. The day 6 solution taken from both pH 7.4 (gray) and pH 4.0 (purple) samples were tested against the HIV-1 pseudo virus PVO.4 in TZM-bl cells. Left: the solution from Silk–HPMC–Glycerol (no Grft). Middle: Solution from Silk–HPMC–Glycerol–Grft from Day 6. Right: TZM-bl cells alone without virus.

In order to determine whether the Griffithsin released from Silk–HPMC–Glycerol–Grft discs is functional and capable of HIV inhibition, the day 6 solution taken from both pH 7.4 (mimicking colorectal condition) and pH 4.0 (mimicking vaginal condition) samples were tested against the HIV-1 pseudovirus PVO.4 in TZM-bl cells. The results showed that the Grft released from the disc at both pH 7.4 and pH 4.0 can fully inhibit the HIV pseudovirus (Figure 6). In order to determine the biocompatibility of Silk–HPMC–Glycerol–Grft discs, the day 6 solution taken from the pH 7.4 Silk–HPMC–Glycerol–Grft sustained release experiment was evaluated by an MTT assay upon incubation with HEK 293Ft cells. The results showed that the cell viability of both the sample and the control (pH 7.4 PBS) were the same, which indicates that the Silk–HPMC–Glycerol–Grft has good biocompatibility.

## 4. Discussion

Although advances in antiretroviral therapy (ART) have shown great effectiveness in the suppression of HIV replication and in preventing HIV acquisition, there are still challenges for HIV eradication and the prevention of new infections. There are several choices on the market for pre-exposure prophylaxes (PrEPs), including a daily pill and a recently approved injectable, Apretude (cabotegravir extended-release injectable suspension), which can be given every two months to reduce the risk of HIV acquisition [67]. Other forms of PrEPs have been incorporated into scaffolds to form microbicides, including sponge-like discs, creams, gels, films, intravaginal rings (IVR), and tablets, which can be applied topically to the vagina or rectum to avoid the sexual acquisition of HIV [19,68]. Among these, the dapivirine vaginal ring has shown effectiveness in women over 25 years old, and in 2021, the WHO recommended the ring as an additional prevention option for women; several Africa countries have now approved its usage for the prevention of HIV [69]. While clinical trials have included several types of insertable devices that are likely to be effective if used consistently, results often show low adherence among users, particularly young women, leading to low protection in this group [70,71,72,73,74,75].

Currently, only half of HIV-infected people are receiving anti-retroviral therapy [76], suggesting the existence of a large pool of people who could actively transmit HIV if their partner is not protected. Therefore, there is an urgent need for affordable, easy-to-apply, and long-lasting prevention approaches, especially for young women, who tend to have a lower compliance with the use of pills, gels, and rings in clinical trials [72,77,78,79].

**Figure 7:**
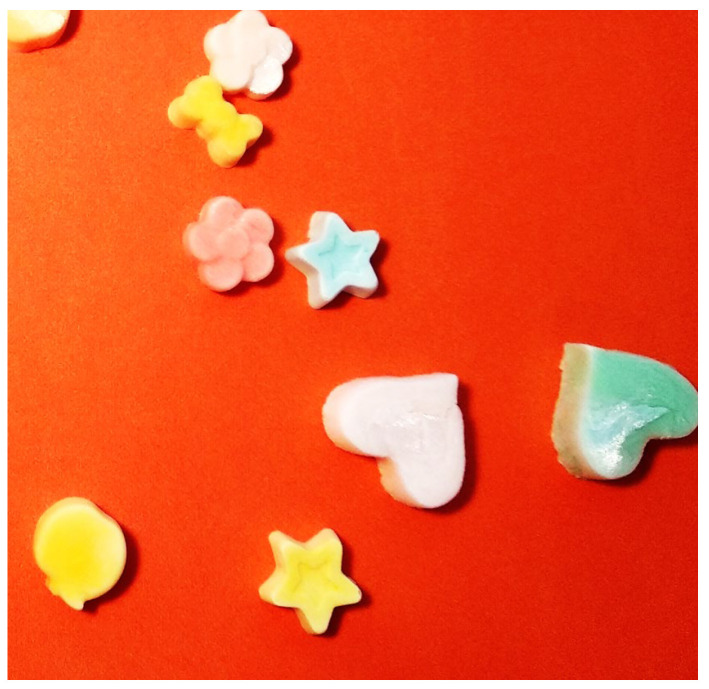
**The silk-based discs can be formed into different shapes and colors can be added**. For this photo, the discs are simply composed of silk fibroin (2%) solution with additional food dye.

We have been working on a silk fibroin-based drug delivery platform that is thermostable (able to be stored without refrigeration) and can be readily applied to vaginal and rectal mucosal sites. We have shown that SF is able to stabilize HIV inhibitory proteins for over a year at high temperatures, and that it can release various proteins over the course of a month [38,39]. Furthermore, in pre-clinical trials in macaques, we showed that SF–Grft discs provide protection to both vaginal and rectal tissue [77]. This platform has the added benefit of being able to be formulated into visually pleasing shapes that also have favorable tactile properties (Figure 7). SF has emerged as a promising drug carrier for bioactive compounds, including small molecules, protein drugs, and antibodies, that has superior biocompatibility and tunable biodegradation [39,80].

Griffithsin is a protein lectin that has been shown to inhibit many viruses, including HIV-1 and SARS-CoV-2. This protein, which is one of the most potent HIV inhibitors with inhibition constants rivalling that of antibodies, binds to the high-mannose region of the HIV-1 gp120 protein to block its entry into host cells [42,44]. It has been used in several preclinical trials, including with gel inserts that showed inhibition of SHIV, HSV-2 and HPV infections in vivo [45,47,48,81,82,83,84,85]. Grft and its variant, Q-Grft, have been shown to be amenable to both the rapid-release and sustained release from silk fibroin discs and various polymer fibers and nanoparticles [38,84,86,87]. Recently, Grft-based microbicides in the form of a gel and a douche (for vaginal and rectal compartments, respectively) have been evaluated in clinical trials and showed no significant adverse events, with the epithelium and CD4+ cell distribution remaining unchanged [81,88,89]. In addition, Grft has been shown to potently inhibit SARS-CoV-2 live virus infection in Vero E6 cells, with a half maximal inhibitory concentration (IC50) of 63 nM [90] and 33.2 nM reported [91]. Furthermore, a variant of Grft that is stable to oxidation, Q-Griffithsin, has been formulated into an intranasal spray and is now in clinical I trials to evaluate its safety, tolerability, and pharmacokinetics (ClinicalTrials.gov Identifier: NCT05437029). So far, results show that the Q-Griffithsin intranasal spray is safe and effective, since no dose accumulation effect or systemic exposure was observed, and no severe adverse events have been reported [92].

Our work with SF discs in the past has shown success in both the quick-dissolve and sustained release of Grft in amounts that effectively inhibit HIV infection, indicating that SF–Grft is a promising microbicide candidate that may be complementary to the current PrEP treatment to mitigate inconsistent user adherence. Indeed, in preliminary focus groups, silk discs were well received. However, while the SF discs show many properties that are valuable, including low cost, facile formulation, and stability at high temperature, the lack of sustained adhesion to the mucosal surface could limit their effectiveness.

To enhance the SF discs’ adherence to the mucosal surface, we investigated four water-soluble and biocompatible chemical additives: tannic acid, TLC, 3,4-DHB, and HPMC, which contain multiple hydroxyl groups to improve the SF-based scaffolds’ hydrophilicity, which may improve its adhesion to tissue in a wet environment [93]. Tannic acid is a food additive approved by the FDA [94] and a Silk–Tannic acid gel has been shown to adhere to rabbit liver, heart, and skin [93]. TLC is a siderophore analogue that has shown adhesion on solid ionized silica in aqueous solutions [95]. 3,4-DHB, is a metabolite of some foods, such as olives, white grapes, and green tea, and has been shown to have anti-inflammatory activities [96]. HPMC is a cellulose ether and is a food additive as well as having been shown to have mucoadhesive properties [97]. SF–HPMC electrospun scaffolds have been demonstrated to have excellent biocompatibility for human umbilical vein endothelial cell adhesion and proliferation [98]. Our results showed that Silk–Tannic acid does not form into a useable disc after the freeze-drying process. TLC and 3,4-DHB made acceptable discs but did not improve adhesion to tissue over simply SF alone. The addition of HPMC did enhance the adhesion of silk fibroin discs to tissue without compromising its shape, but the Silk–HPMC disc alone disintegrated in PBS within half an hour. While this is favorable for near-instantly accessible protection, some users may prefer longer-term protection.

There are multiple strategies for tuning the silk fibroin degradation rate, such as adjusting the β-sheet crystallinity, manipulating the molecular weight of the silk polymer, controlling the degree of cross-linking, and doping with other molecules [99]. High molecular weight, more β-sheet crystalline regions, and fibrillar structure at the nanoscale level are associated with a lower degradation rate [99]. Here, we used doping and recrystallization to slow down the disintegration of the Silk–HPMC disc. Recrystallization in this context is a process that transforms α-helices/random coils into highly oriented β-sheet crystals which will decrease the degradation of the silk scaffolds. This can be achieved by processes such as autoclaving and water annealing [99]. PEG, glucose, and glycerol were chosen as doping candidates. PEG is a commonly used additive that is a non-ionic, hydrophilic, biocompatible, and biodegradable polymer that has been approved by the FDA for drug delivery applications since 1990 [100]. Glucose is a commonly consumed food; it has been reported that the addition of glucose into silk fibroin induced SF crystallization and enhanced the flexibility of SF films, promoting the wound-healing process [64]. Glycerol is used widely in skin and cosmetic products and is an ingredient in wound and burn treatments approved by the FDA [94,101,102]. Solid-state NMR analyses indicated that glycerol induces SF β-sheet formation [65]. The results presented here show that the Silk–HPMC–Glycerol discs processed under condition 2 (with annealing at 80 °C before freeze-drying) has the slowest disintegration rate compared with other doping chemicals and the control Silk–HPMC disc.

We then incorporated Grft into the Silk–HPMC–Glycerol formulation to form a Silk–HPMC–Glycerol–Grft disc with condition 2 processing and evaluated its morphology, FTIR spectra, adhesion, and the sustained release of Grft as well the ability of the released Grft to inhibit single-round HIV in vitro. SEM images showed that the cross section of the Silk–HPMC–Glycerol–Grft disc has a porous structure similar to that of Silk–HPMC and Silk–HPMC–Glycerol. FTIR spectra demonstrated that doping with glycerol increased the β-sheet formation in the Silk–HPMC disc and that all the silk-based discs—Silk–HPMC, Silk–HPMC–Glycerol, and Silk–HPMC–Glycerol–Grft—converged to form more β-sheet structures after processing under condition 2.

The discs’ adhesion to tissue from stronger to weaker was found to be Silk–HPMC–Glycerol > Silk–HPMC–Glycerol–Grft > Silk–HPMC > Silk disc. These results were consistent at both pH 7.4 (to mimic a rectal environment) and pH 4.0 (to mimic a vaginal environment). In general, adherence at the lower pH was more robust. When using the disc alone with no drag force, the discs were able to remain on the vertical surface indefinitely. It is also noteworthy that the doping of glycerol not only improves the adhesion of Silk–HPMC disc to the tissue but slows down the disintegration of the disc.

The level of sustained release of Grft from three silk-based discs, Silk–Grft, Silk–HPMC–Grft, and Silk–HPMC–Glycerol–Grft, was measured by BLI. The results showed that all of the discs are able to release substantial amounts of Grft in quantities that are fully inhibitory for at least 7 days, and none of them show a high burst release of Grft in the first hour. Both the lack of a burst and the continuous release of effective amounts indicate that these formulations have excellent properties that could be useful in a therapeutic device. The discs reported here could be expected to adhere to the mucosal surface within minutes of insertion. While it is not expected that the disc will necessarily remain in place during sexual activity, the released Grft should be protective within hours. As we have shown with more simple SF devices, the Grft released from a silk scaffold (in this case, the Silk–HPMC–Glycerol–Grft discs) remained active, showing full inhibition against a HIV-1 single round virus with the PVO.4 pseudotype in TZM-bl cells. In addition, the MTT assay showed that Silk–HPMC–Glycerol–Grft discs have good biocompatibility, with the same cell survival as with PBS alone. Therefore, due to the urgent need for affordable and acceptable microbicides, the Silk–HPMC–Glycerol–Grft disc presented here is a good candidate, being inexpensive, easy to store without refrigeration, and having high potency against HIV. Additionally, it can be formed into a variety of colorful shapes which may lead to higher adherence for at-risk youth.

## 5. Conclusions

In this study, we present a silk fibroin-based drug delivery system that provides sustained release of Griffithsin as well as showing adhesion to tissue, indicating likely adhesion to mucosal surfaces that are vulnerable to infection. These SF discs are directly relevant for protection against HIV, and the Grft inhibitor has also been shown to be effective against other viruses such as HPV and SARS-CoV-2. The work involves the addition of HPMC to silk fibroin to improve adhesion and the use of glycerol as a doping agent to reduce the degradation process of the Silk–HPMC disc. The resulting discs (Silk–HPMC–Glycerol–Grft) can adhere to sample skin tissue and achieve the sustained release of active Grft for at least 7 days. This formulation could be applied toward making a variety of biologically useful insertable devices or skin patches that can deliver active compounds ranging from small molecules to proteins to mucosal protective microorganisms.

## Data Availability

All the data that support the findings of this study are included within the article.

## Supplementary Materials

The following supporting information can be downloaded at: https://www.mdpi.com/article/10.3390/ma16165547/s1, Supplementary Video S1: Better adhesion of a silk disc when formulated with HPMC.
